# Skull hemophilia pseudotumor: A case report

**DOI:** 10.1515/med-2021-0245

**Published:** 2021-03-22

**Authors:** Kunzhe Lin, Yong Fan, Zhehui Lin, Xiangzhong He, Shaokuan Huang, Fan Zhang

**Affiliations:** Department of Neurosurgery, Affiliated Fuzhou First Hospital of Fujian Medical University, No. 190, Dadao Road, Fuzhou 350009, Fujian, China; Department of Central Laboratory, Affiliated Fuzhou First Hospital of Fujian Medical University, Fuzhou 350009, Fujian, China; Department of Surgery, Fuzhou Taijiang Hospital, Fuzhou 350009, Fujian, China

**Keywords:** skull hemophilia pseudotumor, a case report, MRI, CT

## Abstract

**Background:**

In this study, a rare case with hemophilia pseudotumor in the skull was reported.

**Case presentation:**

The case was a 34-year-old male patient who was admitted to the hospital, with the complaint of dizziness for more than 1 month. The physical examination indicated that the patient was conscious, who could give right answers to the questions. Moreover, there was a bulge on the left frontal-temporal parietal bone. The head CT showed abnormal density lesions on the left frontal-temporal parietal bone, with multiple irregular calcifications within the border. Skull MRI showed a large clump-like mixed signal at the top of the left frontal ridge. After admission, the patient was subjected to complete preoperative preparation and surgical treatment. Neurological navigation was used to determine the extent of skull defect before surgery to make a surgical incision. Clotting factor VIII substitution therapy was used for the intraoperative and postoperative treatments. The lesion was completely removed.

**Conclusions:**

These results suggest that the skull hemophilia pseudotumor has been rarely seen. According to imaging examination, in combination with family history, the diagnosis can be confirmed. If there is no obvious occupying effect, conservative treatment can be tried. On the contrary, if there is an obvious occupying effect, surgical treatment might be effective, and coagulation factor VIII should be supplemented during the perioperative period.

## 1 Background

Hemophilia A and B are inherited bleeding disorders characterized by deficiency or dysfunction of coagulation protein factors VIII and IX, respectively [1]. Hemophilia pseudotumor is a rare complication of hemophilia, which is characterized by repeated hemorrhage and progressive enlargement of hematoma, also accompanied with slow tissue destruction around the hematoma [2]. Hemophilia pseudotumor always occurs in the bone and soft tissues [3]. The patient would also have a history of trauma. Hemophilia pseudotumor that occurs in the skull has been rarely seen. In this study, we reported a rare case with hemophilia pseudotumor in the skull.

## 2 Case presentation

**Figure 1:**
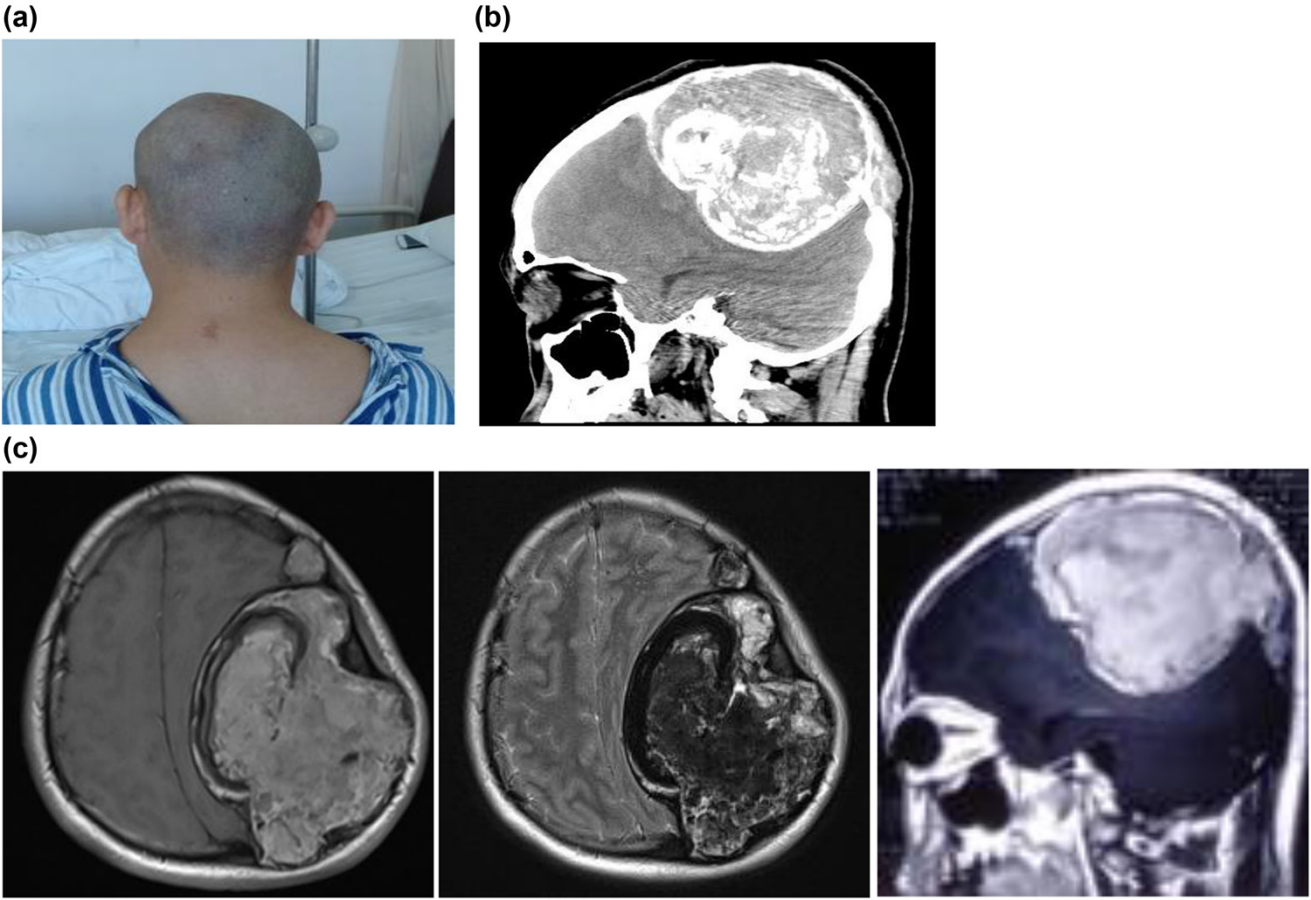
Lesion examination before surgery. (a) Lesion appearance. A bulge on the left frontal-temporal parietal bone was observed, with asymmetry on the left and right sides. (b) Preoperative head CT examination. Abnormal density lesions on the left frontal-temporal parietal bone, with multiple irregular calcifications and clear boundaries were observed. (c) Preoperative MR examination. MRI T1WI: the left frontal lesion showed a slightly higher signal. MRI T2WI: mixed signal. MRI enhanced: visible lesion enhancement.

**Figure 2:**
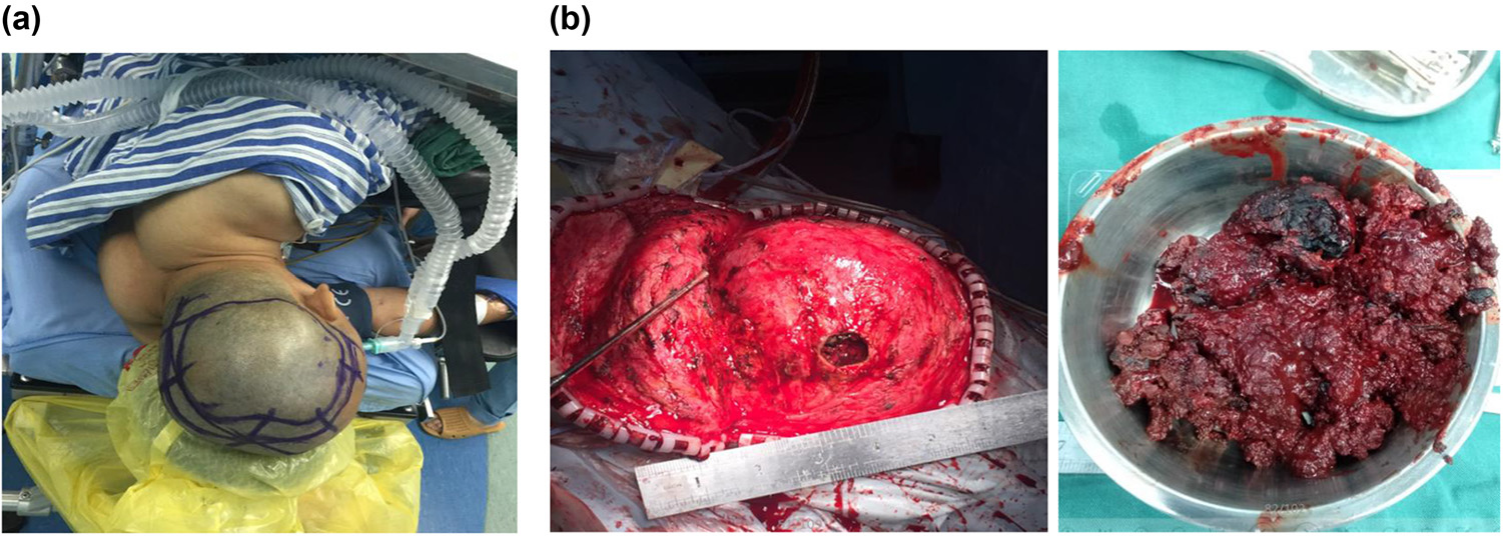
Preoperative observation and intraoperative observation. (a) Nerve navigation was used to detect the skull defect edge to determine the surgical incision. (b) The lesion was observed with complete capsule, containing brown blood clot-like tissue.

**Figure 3:**
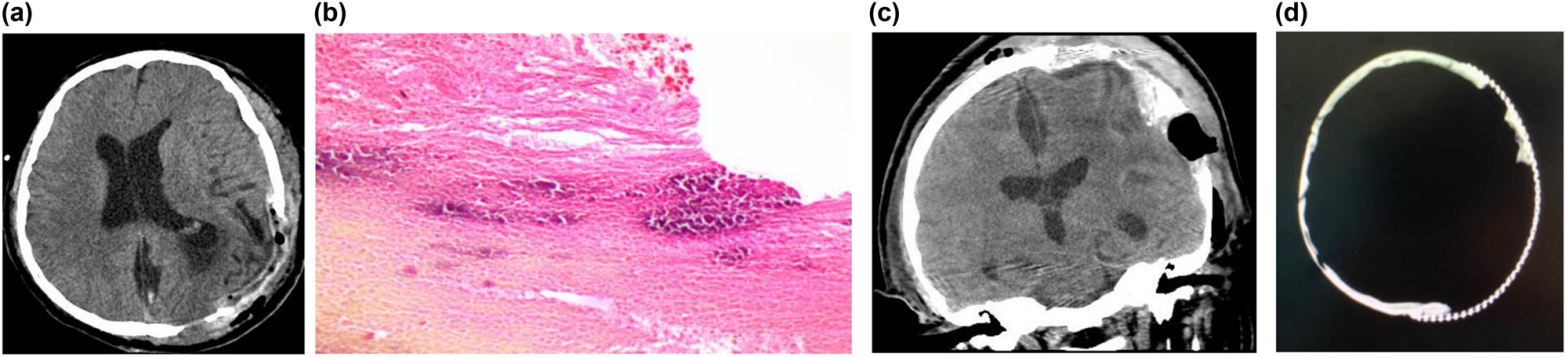
Postoperative examination. (a) CT detection on the first day after surgery. The lesion was completely removed, and the brain tissue was restored. (b) Postoperative pathology. (c) CT detection on the third day after surgery. A small amount of epidural hematoma in the left frontal area occurred, which was conservatively treated. (d) CT detection after cranioplasty at 6 months after surgery.

A 34-year-old male patient was admitted to the hospital, with the complaint of dizziness for more than 1 month. The patient had no obvious neurological deficits except for dizziness. The physical examination indicated that the patient was conscious, who could give right answers to the questions. Moreover, there was a bulge on the left frontal-temporal parietal bone, with a slightly hard texture and no obvious tenderness (Figure 1a). The muscle strength and muscle tension of the patient’s limbs were normal. There were no obvious abnormalities in the cranial nerve examination. For the past history, before about 1 month, the patient was treated for gastric ulcer in a local hospital and had been given the clotting factor VIII. The patient has a family history of hemophilia. The head CT showed abnormal density lesions on the left frontal-temporal parietal bone, with multiple irregular calcifications within the border, as well as clear boundaries (Figure 1b). The skull MRI showed a large clump-like mixed signal at the top of the left frontal ridge, with a slightly higher signal on T1WI and a mixed signal on T2WI, and enhance was observed on contrast-enhanced images (Figure 1c). After admission, the patient was subjected to complete preoperative preparation and surgical treatment. Neurological navigation was used to determine the extent of skull defect before surgery to make a surgical incision (Figure 2a). The lesion was observed with complete capsule, containing brown blood clot-like tissue (Figure 2b). The lesion was completely removed (Figure 3a). After resection, the pathologic examination displayed obvious blood clots and fibrosis hematoma, accompanied by inflammatory cell infiltration and calcification (Figure 3b). On day 3 after surgery, the right upper limb muscle strength was decreased and CT exanimation showed that the patient had an epidural hematoma, which was conservatively treated (Figure 3c). Clotting factor VIII substitution therapy was used for the intraoperative and postoperative treatments. Specifically, at 1 day before and during the operation, coagulation factor 2000U and plasma were supplemented to maintain the level of coagulation factor VIII above 80%. Thereafter, 600–800U coagulation factor was supplemented daily for 2 weeks. The patient was discharged on day 14 after surgery. At discharge, the patient was conscious, with the I/A healing of the surgical incision. The right upper limb muscle strength returned to level 3, and the residual limb muscle strength was level 5. After 6 months, the muscle strength of patient’s limbs was normal. Then, the patient received cranioplasty (Figure 3d).

## 3 Discussion and conclusions

Hemophilia pseudotumor is a chronic, painless, and gradually expanding mass of bone or soft tissue, which has been first reported in 1918 [4]. It usually occurs in the soft tissues, long bones of the lower extremities and pelvis, less in the eyelids, mandible, maxilla, clavicle, humerus, and tibia, only in 1–2% of patients with severe hemophilia and 0–1% of hemophilia patients with normal coagulation function [5]. Hemophilia pseudotumor has been rarely seen in the skull.

In 1972, Kilby et al. [6] have reported a case of hemophilia pseudotumor in the left temporal bone, about 5–6 cm in diameter, who received the surgical treatment. Moreover, Sim et al. [7] have performed surgery on a case of hemophilia pseudotumor in the right parietal bone, with the size of about 5 cm × 5 cm × 2 cm. Furthermore, in 2006, Conde et al. [8] have reported a case of hemophilia pseudotumor located in the left parietal bone, about 5 cm × 3 cm × 3 cm, who received the surgical resection. In 2008, Zafar et al. [9] have reported a case of surgical treatment of right frontal hemophilia pseudotumor, with a diameter of about 3 cm. In the same year, a case of hemophilia pseudotumor with a size of about 20 cm × 16 cm in the right frontal bone has been reported, and the lesion volume has become significantly smaller after the conservative treatment [5].

In 2012, Kashiwazaki et al. [10] have reported a case of progressive enlargement of hemophilia pseudotumor of the left tibia, which has been surgically removed. In 2014, Chen et al. [11] have reported a case of hemophilia pseudotumor located in the right occipital bone, with the size of about 9 cm × 7 cm × 5 cm, which has been subjected to the whole surgical resection and the cranioplasty has been performed on the first stage. In the same year, Xu et al. [3] have reported a case of hemophilia pseudotumor with bilateral parietal and occipital bone, and the case has received surgical treatment. Based on these results, there have been eight cases of pseudotumor tumors of hemophilia in the skull (Table 1), and most of these cases are deficiency for factor VIII. Among them, one case has received conservative treatment and the other seven cases have been subjected to surgical operation. In the case reported in this study, the lesion was located at the frontal-temporal parietal bone, with the size of about 11.3 cm × 8.8 cm × 7.2 cm, which was the case with the largest subjected to surgical treatment.

**Table 1:** Summary of cases of skull hemophilia pseudotumor

First author	Publication year	Lesion location	Lesion size	Treatment plan
Kilby	1972	Left temporal bone	5–6 cm	Surgical treatment
Sim	1996	Right parietal bone	5 cm × 5 cm × 2 cm	Surgical treatment
Conde	2006	Left parietal bone	5 cm × 3 cm × 3 cm	Surgical treatment
Zafar	2008	Right frontal bone	3 cm	Surgical treatment
Inoue T	2008	Right frontal bone	20 cm × 16 cm	Conservative treatment
Kashiwazaki	2012	Left temporal bone	Not described	Surgical treatment
Chen	2014	Right occipital bone	9 cm × 7 cm × 5 cm	Surgical treatment
Xu	2014	Bilateral occipital bone	Not described	Surgical treatment

Hemophilia is an X chromosome-linked disease, and the males have significant advantages. The diseases can be divided into categories A and B, which lack coagulation factors VIII and IX, respectively. The pathogenesis of hemophilic pseudotumor is related to the bone compression, osteoclastic, and new bone formation secondary to and resulting from the intra-articular, soft tissue, or subperiosteal hemorrhage, and bone cortical and intramedullary hemorrhage [11]. In the case reported in this study, the patient had a history of head trauma and did not pay attention to it after the injury. At the time of admission, the patient had no obvious neurological deficits except for dizziness. We speculated that this would be because of the slow growth process of this pseudotumor, so that the patient ignored the abnormal changes in the appearance of the head (covered by the hair). The lesion was accidentally found with the head CT examination. The lesion volume was enlarged at the admission, and the brain midline structure was obviously shifted. Moreover, the brain tissue was significantly compressed, with clear surgical indications.

The treatments of hemophilia pseudotumor include the surgical resection and replacement therapy. It has been recommended to perform surgical resection of cases with progressively enlarged lesions to prevent skin necrosis, rupture, and subsequent bleeding. Surgical treatment can relieve the occupying effect of the lesion in time and repair the skull. It is a radical treatment, but there are risks for complications such as hemorrhage, pseudotumor recurrence, and infection. During the perioperative period, the supplementation of factor VIII is key to ensure the intraoperative and postoperative coagulation.

This patient had an epidural hematoma after surgery. Fortunately, the amount of hematoma was not large, and it was gradually absorbed after the supplement of factor VIII. We believed that the patient’s right upper limb muscle strength decline was not necessarily due to the delayed epidural hematoma, because the hematoma volume was less than 30 mL, with relatively less obvious space-occupying effects. Zhai et al. [4] have reported 23 patients with hemophilia pseudotumor who underwent surgery. Among these patients, three cases have developed wound infection (two of them have had metal implants). Patients with hemophilia pseudotumor occurring in the skull are rarely seen. For patients with large lesions and severe skull erosion, the timing of cranioplasty needs to be further explored. Xu et al. [3] and Chen et al. [11] have performed cranioplasty on the first stage. When the patients with skull hemophilia pseudotumor patients at the first stage were subjected to cranioplasty, postoperative infection will bring serious adverse consequences. In the case reported in this study, the cranioplasty was performed on the second stage. For the patients with hemophilic pseudotumor tumors with less significant occupying effect, factor VIII replacement therapy can be selected, and the cases should be closely followed. Although the case had a hemophilia pseudotumor in the right frontal part, with the size of 20 cm × 16 cm, the main lesion was located under the scalp, and only a small part was located in the cranium. After conservative treatment, the lesion volume had been significantly reduced.

Skull hemophilia pseudotumor has been rarely seen. According to imaging examination, in combination with family history, the diagnosis can be confirmed. If there is no obvious occupying effect, conservative treatment can be tried. Once bleeding occurs, factor substitution can minimize the furthermore damage and reduce the hematoma [12]. On the contrary, if there is an obvious occupying effect, surgical treatment might be effective, and coagulation factor VIII should be supplemented during the perioperative period.
